# Identification of High-Confidence Structural Variants in Domesticated Rainbow Trout Using Whole-Genome Sequencing

**DOI:** 10.3389/fgene.2021.639355

**Published:** 2021-02-25

**Authors:** Sixin Liu, Guangtu Gao, Ryan M. Layer, Gary H. Thorgaard, Gregory D. Wiens, Timothy D. Leeds, Kyle E. Martin, Yniv Palti

**Affiliations:** ^1^National Center for Cool and Cold Water Aquaculture, Agricultural Research Service, United States Department of Agriculture, Kearneysville, WV, United States; ^2^BioFrontiers Institute, University of Colorado Boulder, Boulder, CO, United States; ^3^Department of Computer Science, University of Colorado Boulder, Boulder, CO, United States; ^4^Center for Reproductive Biology, School of Biological Sciences, Washington State University, Pullman, WA, United States; ^5^Troutlodge Inc., Sumner, WA, United States

**Keywords:** rainbow trout, structural variants, copy number variants, transposable elements, repetitive sequence, whole-genome sequencing

## Abstract

Genomic structural variants (SVs) are a major source of genetic and phenotypic variation but have not been investigated systematically in rainbow trout (*Oncorhynchus mykiss*), an important aquaculture species of cold freshwater. The objectives of this study were 1) to identify and validate high-confidence SVs in rainbow trout using whole-genome re-sequencing; and 2) to examine the contribution of transposable elements (TEs) to SVs in rainbow trout. A total of 96 rainbow trout, including 11 homozygous lines and 85 outbred fish from three breeding populations, were whole-genome sequenced with an average genome coverage of 17.2×. Putative SVs were identified using the program Smoove which integrates LUMPY and other associated tools into one package. After rigorous filtering, 13,863 high-confidence SVs were identified. Pacific Biosciences long-reads of Arlee, one of the homozygous lines used for SV detection, validated 98% (3,948 of 4,030) of the high-confidence SVs identified in the Arlee homozygous line. Based on principal component analysis, the 85 outbred fish clustered into three groups consistent with their populations of origin, further indicating that the high-confidence SVs identified in this study are robust. The repetitive DNA content of the high-confidence SV sequences was 86.5%, which is much higher than the 57.1% repetitive DNA content of the reference genome, and is also higher than the repetitive DNA content of Atlantic salmon SVs reported previously. TEs thus contribute substantially to SVs in rainbow trout as TEs make up the majority of repetitive sequences. Hundreds of the high-confidence SVs were annotated as exon-loss or gene-fusion variants, and may have phenotypic effects. The high-confidence SVs reported in this study provide a foundation for further rainbow trout SV studies.

## Introduction

Structural variants (SVs) refer to sequence variants greater than 50 bp in size (Alkan et al., 2011; Ho et al., 2020). The most common SVs include deletions, duplications, inversions, insertions and translocations. SVs are a major source of genetic variation, and affect a larger proportion of the human genome than single nucleotide polymorphism (SNP) and any other genetic variants (Sudmant et al., 2015). SVs can influence the expression of genes (Chiang et al., 2017; Spielmann et al., 2018), and impact numerous phenotypes in both plant and mammals (Spielmann and Mundlos, 2013; Deane-Coe et al., 2018; Zhou et al., 2019; Alonge et al., 2020; Liu et al., 2020). Therefore, it is essential to study SVs in order to explore the full spectrum of genetic variation.

Structural variants are poorly characterized in most species due to both financial and technical constraints. Only a few SV studies were reported in aquaculture species. Based on whole-genome sequences of 492 Atlantic salmon (*Salmo salar L.*), 15,483 high-confidence SVs were identified (Bertolotti et al., 2020). Using a combination of reduced representation sequencing and sequence capture enrichment, SNPs and SVs were identified in American lobster (*Homarus americanus*) (Dorant et al., 2020). This study also reported that 46 SVs were significantly associated with the annual variance of sea surface temperature while SNPs failed to reveal population adaption to local environments.

Repetitive sequences, especially transposable elements (TEs), are a major component of plant and animal genomes (Lisch, 2013; Sotero-Caio et al., 2017), and TEs are an important source for SVs (Payer and Burns, 2019). Initially, specialized microarrays were used to detect genome-wide TE insertions in the human genome (Huang et al., 2010). With the recent advancement of genome sequencing technology, both short-reads and long-reads can be used to detect TE insertions (Zhou et al., 2020; Zook et al., 2020). Computer programs specialized for the detection of mobile element insertions (MEIs) from whole-genome sequences have been developed (Chu et al., 2020), and were used to detect MEIs in many species such as human (Sudmant et al., 2015), grape (Zhou et al., 2019), rice (Kou et al., 2020), and tomato (Alonge et al., 2020; Dominguez et al., 2020).

Rainbow trout (*Oncorhynchus mykiss*) is one of the most widely cultured cold freshwater fish, with production on every continent except Antarctica (Food and Agriculture Organization (FAO), 2021). SVs are a largely unexplored feature in rainbow trout. Classical cytogenetic studies revealed chromosomal number variation from 2N = 58 to 2N = 64 in rainbow trout (Thorgaard, 1983). Recently, comparison of the *de novo* genome assembly and SNP linkage maps revealed a 55 Mb double inversion associated with a migratory trait in rainbow trout on chromosome Omy05 and another 14 Mb inversion on chromosome Omy20 (Pearse et al., 2019). However, a systematic study of SVs in rainbow trout has not been reported to date. The objectives of this study were 1) to identify and validate SVs in rainbow trout using whole-genome re-sequencing; and 2) to examine the contribution of TEs to SVs in rainbow trout. The high-confidence SVs reported in this study are useful resources to understand and explore the genetic variation in rainbow trout, and provide a foundation for further rainbow trout SV studies.

## Materials and Methods

### Ethics Statement

The fish used in this study were sampled according to our standard operating procedures of care and use of research animals, approved by the Institutional Animal Care and Use Committee, National Center for Cool and Cold Water Aquaculture, Agricultural Research Service, United States Department of Agriculture, United States of America. All efforts were made to minimize suffering and to ensure fish welfare.

### Whole-Genome Sequencing and Read Mapping

Three groups of rainbow trout (Supplementary Table S1), including 11 double haploid (DH) lines, 39 outbred fish from the National Center for Cool and Cold Water Aquaculture (NCCCWA) (Leeds et al., 2010, 2016; Wiens et al., 2018) and 46 outbred fish from the Troutlodge May spawning (TLUM) population, were used in this study. DNA was extracted from fin clips following the manufacturer’s recommended protocols for AutoGenprep 965 (Autogen, Holliston, MA, United States). Sequencing and read mapping of the 11 DH lines were described in our previous study (Gao et al., 2018). For both the NCCCWA and TLUM samples, whole-genome DNA sequencing libraries were prepared using the KAPA HyperPrep kit (KAPA Biosystems, Wilmington, MA, United States), and were sequenced in paired-end (2 × 150 bp) mode on an Illumina HiSeq X sequencer. The sequence reads were mapped to the rainbow trout reference genome GCF_002163495.1 (Pearse et al., 2019) using BWA-MEM algorithm (Li, 2013), and alignments were converted to BAM (Binary sequence Alignment/Map) format using SAMtools v1.11 (Li et al., 2009). PCR duplicates were marked and removed using Picard^1^ v2.18.2. Only reads mapped to the 29 chromosomes were used for SV detection as described below, and scaffolds that cannot be assigned to the 29 chromosomes and mitochondria sequence were excluded from SV detection.

### SV Detection and Genotyping

We chose to use LUMPY (Layer et al., 2014) to detect SVs in this study because it integrates multiple signals across multiple samples to detect SVs, and several associated tools, as mentioned below, are available to facilitate SV detection. To reduce false positive SV calls, reads overlapping with problematic regions, including extreme high read coverage regions, low complexity regions and gaps in the reference genome, were excluded from SV detection. To identify extreme high-read coverage regions, mosdepth v0.2.9 (Pedersen and Quinlan, 2018) was used to estimate read coverage using the quantize option, and regions with coverage equal or above 200× were converted into a BED (Brower Extensible Data) file. RepeatMasker v4.1.0 (Smit et al., 2020) was used to mask the reference genome GCF_002163495.1, and the low complexity regions were extracted from the output file of RepeatMasker. The low complexity regions separated by less than 1,000 bp were merged using Bedtools v2.27.1 (Quinlan and Hall, 2010), and intervals greater than 200 bp were then retained. The BED file for gaps in the reference genome was generated using a Python script (Bertolotti et al., 2020). The BED files for the three problematic regions were combined, and regions separated by less than 1,000 bp were merged using Bedtools. As recommended in the user instructions of LUMPY, program Smoove^2^ v0.2.5, which integrates the best practices of LUMPY and other associated tools, was used to detect SVs. We followed the four SV calling steps at the population level as described in the Smoove user guide. Briefly, SVs were called initially in each sample, and then SVtools v0.5.1 (Larson et al., 2019) was used to merge the SV calls across all samples. The SVs were genotyped using SVtyper v0.7.1 (Chiang et al., 2015) and annotated with read coverage using Duphold v0.2.1 (Pedersen and Quinlan, 2019).

To identify high-confidence SVs, we used the following steps to filter the putative SVs detected above: 1) SVs with size less than 300 bp were removed using BCFtools v1.9; 2) SVs overlapping with the extreme high-read coverage (equal or greater than 200×) regions or the gaps of the reference genome were excluded using Bedtools; 3) SVs were filtered based on Duphold annotation. Deletions with DHFFC (fold change for the variant depth relative to flanking regions) > 0.7, duplications with DHFFC < 1.3 and inversions with DHFFC > 1.3 or DHFFC < 0.7 in at least one samples were excluded using BCFtools; 4) SVs with a heterozygous genotype in at least one of the 11 DH lines were also excluded; 5) Variant allele frequencies (AF) were calculated using SVtools subcommand afreq, and SVs with AF < 0.05 or AF > 0.95 were excluded; 6) Genotype call rate for each remaining SV was calculated using the computer program KING v2.2.5 (Manichaikul et al., 2010), and SVs with genotype call rates less than 0.8 were filtered out.

To examine the repetitive DNA content of the SV regions, we extracted the SV sequences from the reference genome GCF_002163495.1 using Bedtools. The rainbow trout repetitive sequence database reported by Pearse et al. (2019) was used to mask the SV sequences using RepeatMasker v4.1.0 with default parameters.

### SV Validation Using Long-Reads

Deep-coverage (111.6×) Pacific Biosciences (PacBio) long-reads of the Arlee homozygous line (unpublished data) were used to validate the high-confidence SVs present in the Arlee line. The long-reads were mapped to the reference genome GCF_002163495.1 using minimap2 v2.17-r941 (Li, 2018). The Python scripts and methods reported by Abel et al. (2020) were then used for SV validation. Briefly, the Python scripts were used to generate a BEDPE (paired-end BED) file to represent the breakpoints of split reads (based on alignment with the reference genome) and reads with insertion or deletion (based on cigar strings) larger than 50 bp, respectively. To support a putative SV call, the long-reads should have breakpoints overlapping with the putative SVs based on Bedtools (“pairtopair -type both -sloop 100 -is” or “intersect -r -f 0.9”). Also, the long-reads and the putative SVs should have the same type of breakpoints such as deletions or duplications. Only putative SVs supported by at least two long-reads were considered as validated SVs.

### Principal Component Analysis

The SV genotypes of the NCCCWA and TLUM fish were used for principal analysis using PLINK v1.9 (Chang et al., 2015) with the top 10 principal components. The 11 DH lines were excluded from the principal component analysis because they were derived from diverse populations.

### SV Annotation

We used SnpEff v5.0 (Cingolani et al., 2012) to annotate the high-confidence SVs. The GTF file of the rainbow trout reference genome, GCF_002163495.1, was downloaded from NCBI, and was used to build a reference database. The high-confidence SVs were then annotated using the default parameters of SnpEff.

## Results

### Identification and Validation of SVs in Rainbow Trout

**FIGURE 1:**
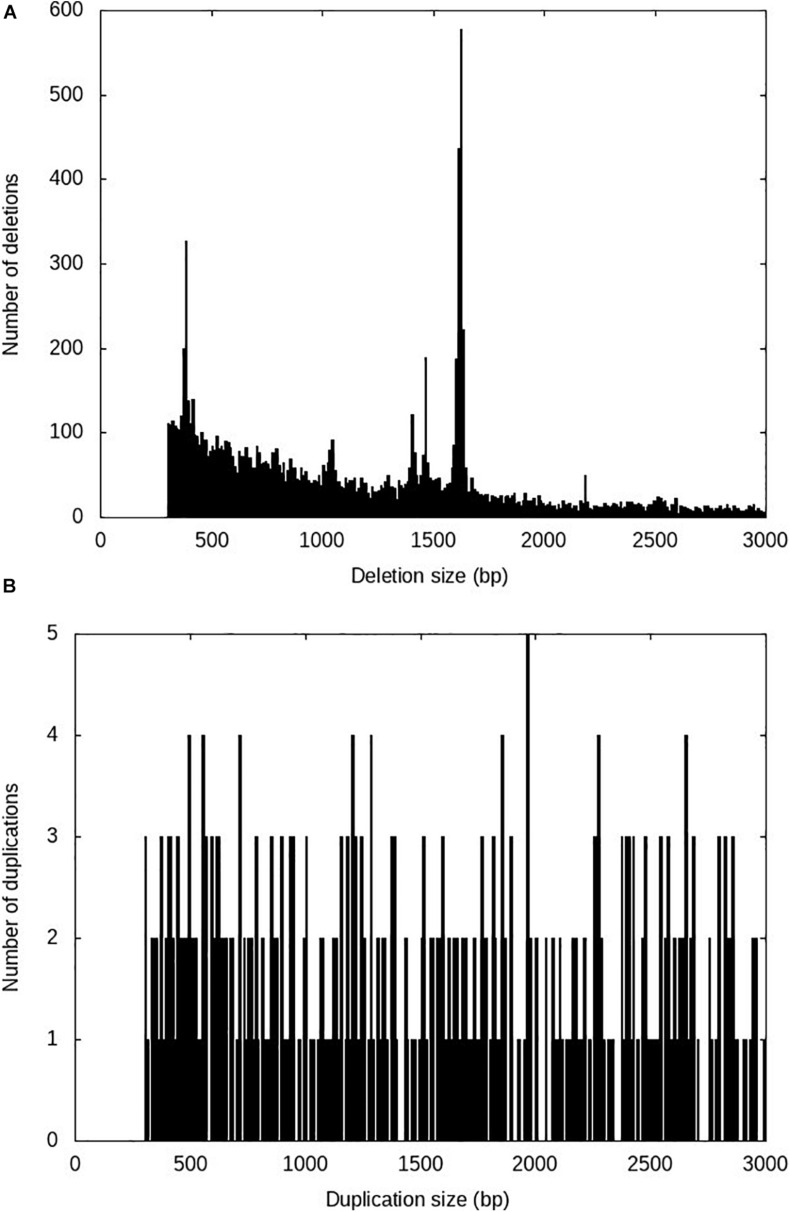
Size distributions of the high-confidence SVs identified in rainbow trout. **(A)** Deletions; **(B)** Duplications.

After running the four SV calling steps at the population level using Smoove, a total of 298,410 putative SVs including 253,325 deletions, 37,648 duplications and 7,434 inversions were identified among the 96 fish used in this study. After rigorous filtering as described in the methods, 13,863 high-confidence SVs including 13,138 deletions, 719 duplications and 6 inversions were retained (Table 1). The average spacing across all chromosomes was 126.6 kb per high-confidence SV (Table 1). The number of high-confidence SVs per sample ranged from 2,618 to 5,924 (Supplementary Table S1) with a median of 5,283 high-confidence SVs per sample. The SV sizes ranged from 300 to 27,641 bp, and 90% of the SVs are shorter than 3,000 bp. The SV size distributions for deletions and duplications are shown in Figures 1A and B, respectively.

**TABLE 1:** Number of the high-confidence SVs identified in rainbow trout.*

Chromosome	Total	Average spacing (kb/SV)	DEL*	DUP	INV
1	644	121.8	607	36	1
2	624	127.1	587	37	0
3	520	145.1	500	20	0
4	685	114.0	641	43	1
5	689	120.8	658	31	0
6	553	134.8	530	23	0
7	576	125.4	536	40	0
8	612	125.0	585	27	0
9	549	111.7	519	30	0
10	509	127.8	474	35	0
11	604	120.1	572	32	0
12	647	125.6	613	34	0
13	236	236.8	228	8	0
14	471	147.4	454	17	0
15	450	126.0	426	24	0
16	520	122.4	495	24	1
17	540	128.2	515	25	0
18	406	133.8	385	21	0
19	437	122.7	400	37	0
20	403	93.9	384	18	1
21	299	150.2	286	13	0
22	503	89.5	483	19	1
23	395	117.5	371	23	1
24	263	132.8	249	14	0
25	559	131.2	532	27	0
26	196	180.6	183	13	0
27	317	125.0	300	17	0
28	335	112.1	317	18	0
29	321	120.7	308	13	0
Total	13,863	126.6	13,138	719	6

*DEL, deletions; DUP, duplications; INV, inversions.

Among the 13,863 high-confidence SVs identified, 4,030 SVs were homozygous for the non-reference alleles in the Arlee line. We used the PacBio long-reads of the Arlee line to validate the high-confidence SVs, and 3,948 SVs (98.0%) were validated (Table 2).

**TABLE 2:** Number of validated SVs using long-reads of the Arlee homozygous line.

SV type*	Number of SVs	Validated by indel reads	Validated by split reads	Number of validated SVs	Validation rate
DEL	3,972	3,589	3,139	3,897	98.1%
DUP	58	NA	51	51	87.9%
Total	4,030	3,589	3,190	3,948	98.0%

*DEL, deletions; DUP, duplications.

### Substantial TE Contribution to SVs in Rainbow Trout

Several peaks were observed in the size distribution of the high-confidence deletions (Figure 1A). The tallest peak was in the 1,610–1,630 bp size range. Among the 1,072 sequences in this size range, 1,030 (96%) sequences had high sequence similarity (identity > 95%) with the full sequence of omyk_TCE_37 (sequence length 1,621 bp, Supplementary Note), the most abundant Tc1-mariner transposon family in rainbow trout (Pearse et al., 2019). This deletion size spike was not unexpected as the Tc1-mariner family omyk_TCE_37 has been active recently and makes up more than 2% of the rainbow trout genome (Pearse et al., 2019).

The next most prominent peak was in the 370–390 bp size range. There were 528 high-confidence deletions in this size range. BLASTN searches against the repeat database of rainbow trout (Pearse et al., 2019) revealed that 250 (47%) of those sequences aligned well (identity > 80%) with the long terminal repeat (LTR) of the Gypsy retrotransposon Omy_1322 (Supplementary Note). The third most prominent peak was in the 1,460–1,470 bp size range. Among the 181 deletion sequences in this size range, 148 (82%) of those sequences were homologous to an unclassified TE sequence (Supplementary Note) in rainbow trout.

Since most sequences in the three peak regions were homologous to TE sequences in rainbow trout, we decided to examine the proportion of repetitive sequences among all high confident SVs reported in this study. As shown in Table 3, 88.3% nucleotides of the deletions were masked by RepeatMasker. For the duplications and inversions, 73.0 and 74.0% nucleotides were masked, respectively. Overall, 86.5% nucleotides of the high-confidence SVs were masked by RepeatMasker, which is much higher than 57.1% repetitive DNA content in the rainbow trout genome (Pearse et al., 2019). Since most repetitive sequences were TEs, TEs contributed substantially to the high-confidence SVs reported in this study.

**TABLE 3:** Repetitive DNA content of the high-confidence SV sequences.*

SV type*	DEL	DUP	INV	All
Number of SVs	13,138	719	6	13,863
Total length (bp)	19,209,805	2,605,465	47,591	21,862,861
Masked (%)	88.3%	73.0%	74.0%	86.5%

*DEL, deletions; DUP, duplications; INV, inversions.

### Principal Component Analysis

**FIGURE 2:**
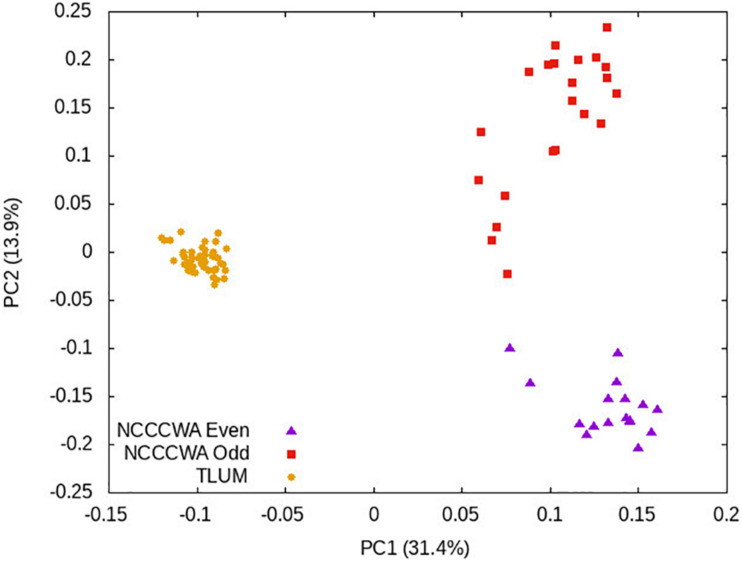
Scatter plot of the 85 outbred fish based on the top two principal components. TLUM fish are represented by yellow dots. Red squares and purple triangles represent the NCCCWA odd-year and even-year fish, respectively.

True SVs can capture the population structure of animals (Bertolotti et al., 2020) and plants (Zhou et al., 2019). The 85 outbred fish used in this study were selected from three distinct rainbow trout populations, NCCCWA even-year population, NCCCWA odd-year population and Troutlodge odd-year May spawning population. Principal component analysis was performed to test whether we can group the fish into three clusters consistent with their populations of origin. As shown in Figure 2, the first principal component clearly separated the Troutlodge population from the two NCCCWA populations, and the second principal component separated the two NCCCWA year-class populations. This result further confirm that the high-confidence SVs reported in this study are robust.

### Annotation of the High-Confidence SVs in Rainbow Trout

The computer program SnpEff (Cingolani et al., 2012) was used to annotate the high-confidence SVs reported in this study. Four impact groups, high, moderate, low and modifier, were used to annotate the putative SV effects. Although 95.53% of the SV effects fell into the modifier group, 3.43% of the SV effects were in the high impact group, and 0.15% SVs were annotated with moderate effect. The rest 0.89% of the SV effects were in the low impact group. Hundreds of SVs were annotated as exon loss variants (1.5%) or gene fusion variants (0.4%) although most SVs were annotated as intron variants (45.5%) or intergenic variants (25.3%) (Supplementary Table S2). Therefore, the high-confidence SVs reported in this study provide a useful genomic resource for further investigation of SV impacts on phenotypes in rainbow trout.

## Discussion

### SVs in Rainbow Trout

Structural variants are a major source of genetic and phenotypic variation but are largely unexplored in many species, including rainbow trout. In this study, 13,863 high-confidence SVs were identified in rainbow trout using whole-genome sequencing. Both the high SV validation rate and principal component analysis indicated that the high-confidence SVs are robust. Although most SVs are in introns and intergenic regions, hundreds were annotated as high impact variants. Thus, the high-confidence SVs reported in this study provide a useful genomic resource for future SV studies in rainbow trout. Moreover, the outbred fish used in this study were from three breeding populations. Therefore, the high-confidence SVs reported in this study are relevant for the identification of SVs associated with traits targeted by selective breeding. However, there are some limitations with the approaches used in this study, and should be improved in the further studies. Although LUMPY is a widely used SV callers, it has some limitations regarding to SV sensitivity and accuracy just like any other SV callers (Layer et al., 2014; Cameron et al., 2019). Also, rigorous filtering steps were used to remove false-positive SVs, some true SVs might be filtered out. Furthermore, DNA sequencing technologies and SV detection algorithms are evolving rapidly (Ho et al., 2020). Thus, the full SV spectrum in rainbow trout remains to be explored.

Each DH line is formed from the duplication of a single gametic cell and hence contains two nearly identical copies of maternal or paternal genome (Parsons and Thorgaard, 1985). It is not surprising that more high-confidence SVs were obtained in each outbred fish (ranged from 4,809 to 5,924) than in each DH line (ranged from 2,618 to 4,149) (Supplementary Table S1). The SVs in each DH line were derived from only one haploid genome, whereas the SVs in each outbred diploid fish came from both the maternal and paternal genomes. The number of SVs in each DH line is largely determined by its genetic distance from the Swanson DH line, which was used for the rainbow trout reference genome assembly. Over 4,000 SVs were observed in each of the Arlee, Hot creek, OSU and Golden lines, which is much more than the number of SVs in each of the other DH lines (2,618–3,783). This observation is consistent with the previously published phylogenic tree of the DH lines based on SNP genotypes (Palti et al., 2014). The four DH lines are more distantly related to the Swanson line than the other DH lines used in this study.

It is essential to remove false positive SVs to retain only high-confidence SVs (Cameron et al., 2019; Ho et al., 2020). Among the 4,030 high-confidence SVs present in the Arlee DH line, 98% SVs were validated using PacBio long-reads. The high SV validation rate is likely due to multiple reasons. Both read mapping and SV detection are much easier for the Arlee DH line, which contains only homozygous genotypes. Also, it is unlikely that true SVs could not be validated due to a lack of long-read coverage in the SV regions because deep genome coverage (111.6×) of the PacBio long-reads were used for SV validation. Additional factors also contributed to this high SV validation rate. First, extreme high read coverage regions and low complexity regions were excluded from SV discovery as described in the section “Materials and Methods,” and putative SVs spanning extreme high read coverage regions were also removed from the putative SVs. The high read coverage regions are known for a high false discovery rate (FDR), which was as high as 99.2% in Atlantic salmon (Bertolotti et al., 2020). Second, the program Smoove, which was used to detect and filter SVs, integrates LUMPY, a widely used SV caller, and Duphold, a program to annotate SVs with the depth of sequence coverage, into one package. As described in the section “Materials and Methods,” low-confidence SVs were filtered out using the sequence depth annotation provided by the program Duphold. Third, additional stringent filtering steps were used to exclude low-confidence SVs. Because small size SVs have higher FDR (Cameron et al., 2019), putative SVs smaller than 300 bp were removed from the dataset. Furthermore, SVs with heterozygous genotypes in any of the 11 DH lines were removed. At last, SVs with AF < 0.05 or AF > 0.95 or SVs with genotype call rates <0.8 were also filtered out. Thus, the high-confidence SVs reported in this study are robust, and are useful resources for understanding and exploring the genetic variation in rainbow trout.

### TEs Contribute Substantially to SVs in Rainbow Trout

Repetitive sequences, especially TEs, have been shown to be a major component of genome architecture, diversity and evolution (Lisch, 2013; Sotero-Caio et al., 2017). The rainbow trout genome contains 57.1% repetitive DNA (Pearse et al., 2019). In this study the repetitive DNA content is much higher in the SV sequences as 85.6% of their nucleotides were masked by RepeatMasker. Since most of the repeat sequences are TEs, TEs contribute substantially to SVs in rainbow trout. Consistent with the results of this study, TE enrichment in SVs was also observed in cichlids species (Faber-Hammond et al., 2019; Penso-Dolfin et al., 2020). However, the extent of TE contribution to SVs might vary across species. Although deletions caused by a recently active DNA transposon were observed in Atlantic salmon, 65% of the high-confidence SVs identified in Atlantic salmon contained no repeat sequences (Bertolotti et al., 2020). Therefore, rainbow trout and Atlantic salmon likely have quite different TE content in their high-confidence SV regions despite their close genetic relationship (Macqueen and Johnston, 2014) and similar repetitive DNA content (Lien et al., 2016; Pearse et al., 2019). Among the phase 3 release of human SVs based on the 1000 Genome Project, about 24% SVs belonged to the MEI class (Sudmant et al., 2015). In the latest release of human SVs (Abel et al., 2020), MEIs accounted for 27% SVs. In pig, Zhao et al. (2016) reported that 54.4% deletions were caused by TEs. In tomato, Alonge et al. (2020) reported that 84% deletions and 76% insertions larger than 100 bp contained at least one TE.

The exact amount of TE contribution to SVs in rainbow trout needs to be further investigated. Due to the technical constraints such as short-reads and read coverage and the SV detection method used in this study, the SV type and size biases are unavoidable. However, the contribution of TEs to SVs in rainbow trout might be underestimated due to the following reasons. First, extreme high read coverage regions which are enriched with TEs were excluded from the SV discovery, and putative SVs spanning these regions were excluded during the SV filtering process. Furthermore, there are still thousands of gaps in the current reference genome assembly (GCF_002163495.1) due in large part to the difficulty to assemble repeat and TE rich regions using Illumina short-reads. Thus, SVs located in the assembly gaps cannot be identified, which likely leads to underestimated TE content of the SV sequences. Second, the program LUMPY used for SV identification cannot detect sequence insertions. So, non-reference MEIs cannot be identified in this study; Third, low frequency SVs were filtered out. It is known that the bulk of MEIs identified in human have low variant allele frequencies (Sudmant et al., 2015; Wildschutte et al., 2016). It was possible that many SVs containing TEs were filtered out due to low allele frequencies; Lastly, long-read DNA sequencing was shown to be more effective than short-reads for detecting SVs that contain TE sequences (Zhou et al., 2020). However, short-reads were used for SV discovery in this study as the short-read sequencing technology is still much more affordable for whole-genome sequencing of multiple samples. Future studies using long-read sequencing technology will likely allow for more accurate quantification and characterization of the impact of TEs on SVs in rainbow trout.

The contribution of TEs to SVs in rainbow trout is not limited to a specific TE class. Both retrotransposon and DNA transposons derived SVs were identified in this study. The most prominent peak with a deletion size range 1,610–1,630 bp (Figure 1A) was due to a DNA transposon. Near half of the deletion sequences in the size range 370–390 bp aligned well with the LTR of Gypsy retrotransposon Omy_1322. These SV sequences were not full-length Gypsy retrotransposons, and they were solo LTRs. Solo LTRs are derived from ectopic recombination between LTRs (Thomas et al., 2018), and about 90% LTR retrotransposons in human genome are represented by solo LTRs (Subramanian et al., 2011). Since we did not observe deletions matching the full length of the Gypsy retrotransposon Omy_1322, we speculate that the majority of this retrotransposon sequences in rainbow trout are solo LTRs. Furthermore, among all the high-confidence SVs reported in this study, 25.5% nucleotides were masked by retrotransposons, 41.2% nucleotides were masked by DNA transposons, and 18.9% nucleotides were masked by unclassified TEs.

## Conclusion

Structural variants are increasingly being recognized for their importance in evolutionary processes and impact on variation of economically important traits in plants and animals. In this paper we report for the first time on the discovery and validation of high-confidence SVs in rainbow trout which provides a foundation for further studies on their impact on phenotypic variation in this species. Our report also includes the finding of substantial TE contribution to SVs in rainbow trout, which provides an opening for further research on the role of TEs in the evolution of salmonid species given the smaller TE content in the high-confidence SVs in Atlantic salmon reported previously.

## Data Availability Statement

The raw sequences of the 85 outbred fish were submitted to NCBI SRA (BioProject PRJNA681179), and the raw sequences of the 11 double haploid lines are also available in NCBI SRA (BioProject PRJNA386519). The PacBio long-reads of Arlee line are available in NCBI SRA under BioProject PRJNA623027. The VCF file for the high-confidence SVs reported in this study was submitted to the European Variation Archive under project PRJEB41774.

## Ethics Statement

The fish used in this study were sampled according to our standard operating procedures of care and use of research animals, approved by the Institutional Animal Care and Use Committee, National Center for Cool and Cold Water Aquaculture, Agricultural Research Service, United States Department of Agriculture, United States of America. All efforts were made to minimize suffering and to ensure fish welfare.

## Author Contributions

SL and YP conceived and planned the experiment. SL performed the data analysis and wrote the manuscript. GG mapped the short-reads to the rainbow trout reference genome. RL and GG contributed to SV identification and validation. GT provided the sequences of the double haploid lines. TL, KM, and GW were involved in selection of the outbred fish used for sequencing. All authors read and approved the final manuscript.

## Conflict of Interest

KM is employed by Troutlodge Inc. The remaining authors declare that the research was conducted in the absence of any commercial or financial relationships that could be construed as a potential conflict of interest.

## Footnotes

1 http://broadinstitute.github.io/picard/

2 https://github.com/brentp/smoove

## Supplementary Material

Supplementary Table 1 Details of the samples used in this study including sample names, populations, genetic lines, read coverage and number of the high-confidence SVs per sample.

Supplementary Table 2 SV effects by type. The high-confidence SVs were annotated using SnpEff.

Supplementary Note The sequences of three transposable elements highlighted in this study.
